# Population-based assessment of factors influencing antibiotic prescribing for adults with dengue infection in Taiwan

**DOI:** 10.1371/journal.pntd.0010198

**Published:** 2022-02-28

**Authors:** Chia-En Lien, Yiing-Jenq Chou, Yi-Jung Shen, Theodore Tsai, Nicole Huang

**Affiliations:** 1 Research Center for Epidemic Prevention, National Yang Ming Chiao Tung University, Taipei, Taiwan; 2 Institute of Public Health, College of Medicine, National Yang Ming Chiao Tung University, Taipei, Taiwan; 3 Office of the Deputy Superintendent, National Yang Ming Chiao Tung University Hospital, Yilan County, Taiwan; 4 Institute of Hospital and Health Care Administration, College of Medicine, National Yang Ming Chiao Tung University, Taipei, Taiwan; 5 Takeda Vaccines, Cambridge, Massachusetts, United States of America; University of Pittsburgh, UNITED STATES

## Abstract

**Background:**

Antibiotic treatment for dengue is likely considerable and potentially avoidable but has not been well characterized. This study aimed to assess antibiotic prescribing for confirmed dengue cases in outpatient and inpatient settings and to identify associated patient, physician and contextual factors.

**Methods:**

57,301 adult dengue cases reported in Taiwan between 2008–2015 were analyzed. We assessed both outpatient and inpatient claims data of dengue patients from a week before to a week after their dengue infections were confirmed under Taiwan’s National Health Insurance program. A multivariable logistic regression with generalized estimating equations was used to estimate the probability of antibiotic prescribing in dengue patients.

**Results:**

Overall, 24.6% of dengue patients were prescribed an antibiotic during the 14 day-assessment period. Antibiotics were prescribed in 6.1% and 30.1% of outpatient visits and inpatient admissions, respectively. Antibiotic prescriptions were reduced by ~50% in epidemic years. Among inpatients, advanced age, females, and major comorbidities were risk factors for receipt of an antibiotic; antibiotics were used in 26.0% of inpatients after dengue was diagnosed. Significant differences in antibiotic prescribing practices were observed among physicians in outpatient settings but not in inpatient settings.

**Conclusions:**

In addition to patient and physician demographic characteristics, contextual factors such as care setting and during epidemics significantly influenced prescription of antibiotics. Characterization of prescribing patterns should help direct programs to curb antibiotic prescribing.

## Author summary

Antimicrobial resistance is a growing global public health threat. The non-specific clinical manifestations of dengue overlap with signs and symptoms of other febrile illnesses common to tropical and subtropical zones making differential diagnosis between dengue and bacterial infections difficult, hence, leading to potentially unnecessary antibiotic prescribing. However, our understanding of factors underlying antibiotic prescribing for dengue is rather limited. Taiwan has experienced periodic dengue outbreaks and has a comprehensive national health insurance database including reliable infectious diseases surveillance and prescribing records. The findings in Taiwan show that other than commonly known patient characteristics, provider and contextual factors play a significant role. Physician’s age and practice setting were significant factors influencing the decision to prescribe antibiotics, particularly in outpatient visits. The likelihood of prescribing an antibiotic to dengue patients was reduced by more than 50% in medical visits occurring after the dengue infection was confirmed. Understanding patient, provider and contextual factors in antibiotics prescription for dengue infections can provide insights for improved antibiotic stewardship and unnecessary antibiotic treatment for dengue.

## Introduction

Growing antimicrobial resistance is recognized as an urgent threat to global health. Among other drivers, such as their promiscuous use in animal husbandry, widespread misuse and overuse of antibiotics in healthcare is a major contributor to the emergence of resistant pathogens [1]. While antibiotics may be prescribed inappropriately for conditions that are likely to have a viral etiology, they also may be used in early intervention of febrile illnesses when the pre-test probability of a bacterial infection argues for empirical therapy. The non-specific clinical manifestations of dengue (DENV) overlap with signs and symptoms of other febrile illnesses common to tropical and subtropical zones, such as leptospirosis, for which antimicrobial therapy is therapeutic [2–7]. At the point of clinical presentation, differentiating dengue from bacterial infections that should be treated with antibiotics may be difficult but also is contextual, depending on the local epidemiology of the respective pathogens [8]. For example, within Thailand, the likelihood of encountering dengue versus leptospirosis differs geographically and pre-test strategies for empirical use of antibiotics in respective locations have been modeled [7,9].

Point-of-care dengue diagnostic assays are not available universally and their performance also depends on the local epidemiology of dengue and other flaviviral infections that can produce cross-reactions. As over half of the global population in at least 120 countries, some four billion people, is at risk for dengue, that antibiotic consumption is likely to be considerable [6].

Compared to a large body of literature describing inappropriate antibiotic prescribing for acute viral respiratory illnesses such as colds, upper respiratory tract infections and bronchitis [9–21], there is a limited understanding of factors underlying antibiotic prescribing for dengue. A few relevant studies have investigated antibiotic use more generally, among acute febrile patients, in dengue endemic regions. Unlike previous reports comprising case series over limited intervals [22,23], this study exploited Taiwan’s national linked databases that covers 99.9% of the population and examined all nationally reported cases over an eight-year period. Like many Asian countries, Taiwan experiences periodic dengue outbreaks and has a high antibiotic prescribing rate [24]. Taiwan’s comprehensive national insurance data and infectious diseases surveillance and reporting system offer a unique opportunity to assess antibiotic prescribing patterns for dengue and associated factors. We analyzed not only patient characteristics, but also physician and contextual characteristics such as in outpatient and inpatient settings, before and after dengue diagnosis and, taking advantage of the periodicity of dengue outbreaks in Taiwan, during an interval of heavy epidemic transmission compared to a quiescent pre-epidemic interval [25]. Furthermore, we also describe the frequency of use for specific antibiotics prescribed during the assessment period.

## Methods

### Ethics statements

This study was reviewed and approved by the Institutional Review Board of National Yang Ming Chiao Tung University (NO. YM109083E).

### Data sources

Since the study was done using administrative datasets without identifiable personal information, individual informed consent was waived. Linked data were obtained from the Notifiable Disease Dataset of Confirmed Cases (NDDCC), operated by the Taiwan Centers for Disease Control (CDC), and the National Health Insurance Research Database (NHIRD) in Taiwan. The Communicable Disease Control Act requires clinicians to report every individual who is infected by or suspected of a notifiable communicable disease, including dengue, to the NDDCC, providing the most accurate source of confirmed dengue cases. The NDDCC was then linked to the NHIRD to obtain medical care utilization information on confirmed cases, including diagnoses, type of medication prescribed, patient and physician characteristics. All these databases are available for research purposes and managed by the Health and Welfare Data Center (HWDC) and linked using encrypted identification numbers of individual patients, physicians, and hospitals. All the analyses were conducted onsite at the HWDC. The validity and quality of the NHIRD have been assessed repeatedly and found to be satisfactory and well-suited for research purposes [26,27].

### Study design and study population

This was a population-based pooled cross-sectional study. The study population included all confirmed dengue cases in persons 18 years and older during the study period, 2008–2015 (60,587 patients). The dengue cases in our sample were defined as dengue cases confirmed by the Taiwan CDC and recorded in the NDDCC dataset. In Taiwan, any suspected dengue case is required to be reported to the Taiwan CDC and his/her specimen also must be sent to Taiwan CDC for confirmation through Taiwan’s official infectious disease surveillance system. The cases confirmed by Taiwan CDC are recorded in the NDDCC dataset. Multiple dengue records of the same individual within 3 months were considered the same dengue case and only the initial report was analyzed. Each dengue case was assessed for his/her utilization of outpatient care, inpatient services, and antibiotics prescribed during a 14-day assessment period (from a week before to a week after the index date). The index date was defined as the individual’s date of confirmed dengue diagnosis recorded in Taiwan CDC’s database. Due to heterogeneity in disease progression, frequency of comorbidities, and factors influencing medical care utilization patterns between adults and children/adolescents, we did not include pediatric populations in our study. People with incomplete information on important characteristics or without any medical visit during the assessment period were excluded (3,286 patients). The final study population included 57,301 dengue cases.

### Variables

Our main dependent variable was the presence or absence of at least one antibiotic prescription for each confirmed dengue case during the 14-day assessment period. Medical utilization patterns included antibiotics prescribed, outpatient visits, and hospitalization events. The medical utilization patterns included any antibiotic prescribed, any outpatient visit incurred, and any hospitalization incurred. All three types of medical service utilization were assessed during the 14-day assessment period. Any antibiotics prescribed, outpatient visit, or hospitalization occurring during the 14-day period, 7 days before confirmation, 7 days after confirmation were assessed. The frequency of specific antibiotics prescribed was calculated. Antibiotics prescribed in medical visits involving a concomitant bacterial infection diagnosis for which antibiotic treatment may be indicated were excluded for analyses [28]. Concomitant bacterial infection was defined as a bacterial infection diagnosis which was recorded in the same claim as dengue diagnosis. We adopted Clinical Classification System, a diagnosis and procedure categorization scheme developed by the Agency for Healthcare Research and Quality, to identify concomitant bacterial infection. We did not restrict the main analyses only to medical visits with a dengue diagnosis because National Health Insurance (NHI) claims for outpatient visits only allow three diagnoses for each visit. According to our data, only 8.1% and 59.2% of the visits shortly before and after dengue was confirmed contained a dengue diagnosis, respectively. Furthermore, we used the Anatomical Therapeutic Chemical (ATC) classification system to identify the type of antibiotics prescribed (ATC code J01). Topical antibiotics were excluded from the analyses.

Patient characteristics included patient’s age, sex, socioeconomic status (SES), comorbidity status, and year of confirmed dengue diagnosis. Patient’s age was classified into three groups: 18–45, 45–65, and above 65 years. SES was defined by the individual’s insurable wages and types. Patients were classified into those with and without regular well-defined monthly wage and the former were then categorized into two groups: monthly wage above or below NTD $30,000 (approximately, $1,000 USD). Comorbidity status was defined by presence of major comorbidities such as cancer, chronic kidney diseases, and others [29]. We also classified year of diagnosis into epidemic dengue (2014 and 2015) and nonepidemic periods.

Physician characteristics included age, sex, specialty, and patient volume. Physician’s age was classified into three groups: under 40, 40–60, and above 60 years. Specialty was classified into five categories: family medicine, internal medicine, emergency, otolaryngology (ENT), and others. Patient volume was calculated separately for outpatient visits and inpatient admissions. Patient volume was defined as the average monthly number of ambulatory care visits or inpatient admissions one year before the index date. Both ambulatory and inpatient volumes were then categorized into tertiles as low, medium, and high. Characteristics of physician’s practice setting included ownership (public vs. private), accreditation level and urbanization of practice (urban vs. rural). We classified medical facilities into three levels: medical centers, regional and district hospitals, and clinics according to Taiwan’s medical facility accreditation regulation and process. Medical centers are the tertiary care hospitals which has met the highest quality of care standard under the current accreditation regulation.

### Statistical analyses

Descriptive statistics were computed to describe the characteristics of dengue patients. Prescribing patterns in outpatient and inpatient settings were analyzed separately. Since a dengue diagnostic test result could have influenced the antibiotic prescribing decision, we analyzed antibiotic prescribing in dengue patients before and after their dengue infection was confirmed. Due to the hierarchical nature of the data, cluster effects are likely; generalized estimating equation (GEE) was applied to control the probable clustering effects of visits within each physician. Robust estimators were applied for standard errors in all analyses. Sensitivity analyses were conducted: (1) instead of analyzing medical visits without a concomitant bacterial diagnosis, we tried different inclusion criteria (i.e. all visits including those with a concomitant bacterial diagnosis; visits with dengue diagnosis, but without a concomitant bacterial diagnosis); (2) a shorter assessment period (6-day period from 3 days before to 3 days after the index date). The results of these sensitivity analyses remained robust. All analyses were conducted using SAS 9.4.

## Results

Of the 57,301 confirmed adult dengue cases during the study period (2008–2015), 89.4% of cases occurred during the 2014–2015 epidemic (Table 1). 21.5% of dengue cases were ≥ 65 years old. Only 7.0% of dengue cases had underlying major comorbid illnesses. 91.6% had fever and 33.7% were hospitalized, of whom 5.4% were admitted to ICUs. In the absence of a documented concomitant bacterial infection, 24.6% received at least one antibiotic during the 14-day assessment period. The average lengths of hospital stay for patients who received antibiotics and who did not receive antibiotics were 8.1 and 6.3 days, respectively (Table A in S1 Text). Table 2 presents distributions of the total and sub-samples at visit-level, and proportions of visits with antibiotic prescriptions by patient and physician characteristics. Cases resulted in a total of 189,510 ambulatory care visits and 18,629 admissions within the 14-day assessment period.

**Table 1:** Patient characteristics and medical utilization patterns among 57,301 confirmed adult dengue patients, Taiwan, 2008–2015.

	Patients with dengue (N = 57,301)
	N (%)
**Epidemic period**	
No	6,079 (10.6)
Yes	51,222 (89.4)
**Patient characteristics**	
Age, y	
18–45	22,866 (39.9)
45–64	22,092 (38.6)
≥65	12,343 (21.5)
Sex	
Male	28,040 (48.9)
Female	29,261 (51.1)
SES	
< $30,000 NTD	21,871 (38.2)
≥ $30,000 NTD	19,391 (33.8)
Others	16,039 (28.0)
Major comorbidities	
No	53,310 (93.0)
Yes	3,991 (7.0)
Fever	
No	4,839 (8.4)
Yes	52,462 (91.6)
**Medical utilization**	
Any antibiotics prescribed	
No	43,190 (75.4)
Yes	14,111 (24.6)
Outpatient utilization	
No	1,263 (2.2)
Yes	56,038 (97.8)
Outpatient utilization before confirmation	
No	17,501 (30.5)
Yes	39,800 (69.5)
Outpatient utilization after confirmation	
No	9,189 (16.0)
Yes	48,112 (84.0)
Hospitalization	
No	38,003 (66.3)
Yes	19,298 (33.7)
Hospitalization before confirmation	
No	54,121 (94.5)
Yes	3,180 (5.5)
Hospitalization after confirmation	
No	41,059 (71.7)
Yes	16,242 (28.3)
ICU utilization	
No	1,041 (5.4)
Yes	18,257 (94.6)

$30 NTD ≌ $1 US dollar.

Abbreviation: SES, socioeconomic status; ICU, intensive care unit.

### Antibiotic prescribing in outpatient settings

Antibiotics were prescribed in 6.1% of outpatient visits during the 14-day assessment period (Table 2). The proportion of visits with antibiotic prescriptions varied slightly across most patient characteristics (5.7%-6.7%). The exception was a relatively larger difference between epidemic (5.8%) and nonepidemic periods (9.3%). In contrast, among physician characteristics, antibiotic prescribing varied considerably by physician’s age, gender, specialty and ownership of practice setting. For outpatient cases, antibiotics were prescribed more often in the week prior to the confirmed date of dengue infection (9.3%) than in the week after the diagnosis was confirmed (4.1%). Similar prescribing patterns across patient and physician characteristics were observed during the pre- and post- dengue confirmation week.

**Table 2:** Outpatient antibiotic use for confirmed dengue by patient and physician characteristics, in pre- and post- dengue diagnosis visits, Taiwan, 2008–2015.

	Total		Pre-dengue confirmation			Post- dengue confirmation		
		Antibiotics use		Antibiotics use			Antibiotics use	
	N (%)	%	N (%)	%	P	N (%)	%	P
**Total visits**	**189,510**	**6.1%**	**75,172**	**9.3%**		**114,338**	**4.1%**	
**Epidemic period**					< .01			< .01
No	19,186 (10.1)	9.3%	11,087 (14.7)	11.3%		8,099 (7.1)	6.5%	
Yes	170,324 (89.9)	5.8%	64,085 (85.3)	8.9%		106,239 (92.9)	3.9%	
**Patient characteristics**								
Age, y					< .01			< .01
18–45	72,612 (38.3)	6.7%	27,996 (37.2)	10.6%		44,616 (39.0)	4.3%	
45–64	75,768 (40.0)	5.7%	29,546 (39.3)	8.7%		46,222 (40.4)	3.7%	
≥65	41,130 (21.7)	5.9%	17,630 (23.5)	8.0%		23,500 (20.6)	4.4%	
Sex					.02			< .01
Male	90,401 (47.7)	5.7%	35,237 (46.9)	9.0%		55,164 (48.2)	3.6%	
Female	99,109 (52.3)	6.5%	39,935 (53.1)	9.5%		59,174 (51.8)	4.4%	
SES					.01			0.87
< $30,000 NTD	72,579 (38.3)	6.3%	28,780 (38.3)	9.7%		43,799 (38.3)	4.1%	
≥ $30,000 NTD	64,934 (34.3)	6.0%	25,508 (33.9)	8.9%		39,426 (34.5)	4.0%	
Others	51,997 (27.4)	6.1%	20,884 (27.8)	9.1%		31,113 (27.2)	8.2%	
Major comorbidity					.08			< .01
No	176,466 (93.1)	6.1%	69,702 (92.7)	9.3%		106,764 (93.4)	4.0%	
Yes	13,044 (6.9)	6.5%	5,470 (7.3)	8.6%		7,574 (6.6)	5.0%	
**Physician characteristics**								
Physician age, Y					.25			< .01
≤40	50,369 (26.6)	5.1%	13,556 (18.0)	9.4%		36,813 (32.2)	3.5%	
40–59	108,827 (57.4)	6.2%	45,207 (60.1)	9.1%		63,620 (55.6)	4.1%	
≥60	30,314 (16.0)	7.7%	16,409 (21.8)	9.6%		13,905 (12.2)	5.6%	
Physician sex					< .01			< .01
Male	167,187 (88.2)	6.4%	68,746 (91.5)	9.5%		98,441 (86.1)	4.3%	
Female	22,323 (11.8)	3.8%	6,426 (8.5)	6.7%		15,897 (13.9)	2.6%	
Physician specialty					< .01			< .01
Family medicine	38,969 (20.6)	3.8%	16,185 (21.5)	6.1%		22,784 (19.9)	2.1%	
Internal medicine	62,606 (33.0)	3.3%	20,806 (27.7)	5.6%		41,800 (36.6)	2.2%	
Otolaryngology	21,511 (11.4)	15.5%	12,870 (17.1)	18.4%		8,641 (7.6)	11.2%	
Emergency	35,567 (18.8)	8.3%	9,238 (12.3)	15.0%		26,329 (23.0)	6.0%	
Others	30,857 (16.3)	5.8%	16,073 (21.4)	6.6%		14,784 (12.9)	4.9%	
Average monthly volume					< .01			< .01
Low	62,536 (33.0)	5.6%	13,688 (18.2)	11.9%		48,848 (42.7)	3.9%	
Medium	62,540 (33.0)	5.9%	27,475 (36.5)	8.6%		35,065 (30.7)	3.8%	
High	64,434 (34.0)	6.8%	34,009 (45.2)	8.8%		30,425 (26.6)	4.6%	
Ownership of practice setting					.03			< .01
Private	149,954 (79.1)	6.7%	68,272 (90.8)	9.4%		81,682 (71.4)	4.5%	
Public	39,556 (20.9)	4.0%	6,900 (9.2)	8.6%		32,656 (28.6)	3.0%	
Accreditation level of practice setting					< .01			< .01
Medical center	34,402 (18.2)	5.2%	6,935 (9.2)	9.8%		27,467 (24.0)	4.0%	
Regional/District hospital	60,735 (32.0)	4.8%	14,972 (19.9)	10.0%		45,763 (40.0)	3.0%	
Clinics	94,373 (49.8)	7.4%	53,265 (70.9)	9.0%		41,108 (36.0)	5.2%	
Urbanization of practice					.05			.02
Urban	158,982 (83.9)	6.0%	59,423 (79.0)	9.4%		99,559 (87.1)	4.0%	
Non-urban	30,528 (16.1)	6.7%	15,749 (21.0)	8.9%		14,779 (12.9)	4.4%	

$30 NTD ≌ $1 US dollar.

Abbreviation: SES, socioeconomic status; P, P-value.

After adjusting for other characteristics and clustering effects, prescribing patterns were relatively consistent for total visits, pre-confirmation visits and post-confirmation visits (Table 3). Significant associations with receipt of antibiotics during the 14 day assessment period were found for patient’s age, gender, and comorbidity status: patients 45–64 years old were significantly less likely to be prescribed antibiotics than patients <45 years (odds ratio [OR]: 0.89; 95% CI: 0.85–0.93); female patients (OR: 1.14; 95% CI: 1.10–1.18) and patients with a major comorbidity (OR: 1.15; 95% CI: 1.07–1.24) were significantly more likely to be prescribed antibiotics than their counterparts. As described above, the likelihood of antibiotic prescribing was significantly lower during epidemic compared to the non-epidemic years (OR: 0.67; 95% CI: 0.61–0.72).

**Table 3:** Patient and physician characteristics associated with antibiotic prescribing for dengue patients in outpatient settings, Taiwan, 2008–2015.

	Total		Pre-dengue confirmation		Post-dengue confirmation	
	OR (95%CI)	AOR (95% CI)	OR (95%CI)	AOR (95% CI)	OR (95%CI)	AOR (95% CI)
**Epidemic period**						
No	Ref	ref	ref	ref	ref	ref
Yes	0.70 (0.64–0.76)	0.67 (0.61–0.72)	0.84 (0.77–0.93)	0.82 (0.74–0.89)	0.65 (0.57–0.73)	0.59 (0.53–0.67)
**Patient characteristics**						
Age, y						
18–45	Ref	ref	ref	ref	ref	ref
45–64	0.89 (0.86–0.93)	0.89 (0.85–0.93)	0.87 (0.82–0.92)	0.89 (0.84–0.93)	0.90 (0.85–0.95)	0.88 (0.83–0.94)
≥65	0.97 (0.90–1.03)	0.99 (0.93–1.06)	0.87 (0.80–0.94)	0.90 (0.84–0.97)	1.03 (0.93–1.13)	1.05 (0.95–1.16)
Sex						
Male	Ref	ref	ref	ref	ref	ref
Female	1.13 (1.08–1.17)	1.14 (1.10–1.18)	1.06 (1.01–1.12)	1.08 (1.02–1.13)	1.21 (1.14–1.28)	1.23 (1.16–1.31)
SES						
< $30,000 NTD	Ref	ref	ref	ref	ref	ref
≥ $30,000 NTD	0.95 (0.91–0.99)	0.98 (0.94–1.02)	0.94 (0.89–1.00)	0.97 (0.92–1.03)	0.97 (0.91–1.03)	0.99 (0.93–1.06)
Others	0.99 (0.95–1.03)	1.01 (0.97–1.06)	0.97 (0.92–1.03)	1.01 (0.95–1.07)	1.00 (0.94–1.07)	1.02 (0.95–1.09)
Major comorbidity						
No	Ref	ref	ref	ref	ref	ref
Yes	1.11 (1.03–1.19)	1.15 (1.07–1.24)	0.98 (0.89–1.08)	1.03 (0.94–1.13)	1.22 (1.10–1.36)	1.27 (1.13–1.42)
**Physician characteristics**						
Physician age, Y						
≤40	Ref	ref	ref	ref	ref	ref
40–59	0.96 (0.86–1.07)	1.06 (0.95–1.18)	0.89 (0.79–1.00)	1.07 (0.94–1.21)	1.05 (0.91–1.20)	1.09 (0.95–1.25)
≥60	1.23 (1.08–1.41)	1.57 (1.36–1.82)	1.03 (0.88–1.19)	1.45 (1.24–1.70)	1.53 (1.27–1.84)	1.83 (1.50–2.24)
Physician sex						
Male	Ref	ref	ref	ref	ref	ref
Female	0.71 (0.61–0.82)	0.87 (0.75–1.01)	0.78 (0.66–0.92)	0.90 (0.75–1.07)	0.70 (0.58–0.84)	0.88 (0.73–1.05)
Physician specialty						
Family medicine	Ref	ref	ref	ref	ref	ref
Internal medicine	0.83 (0.71–0.96)	0.94 (0.81–1.11)	0.94 (0.79–1.12)	1.00 (0.83–1.20)	0.92 (0.76–1.11)	1.00 (0.82–1.22)
Otolaryngology	3.38 (2.84–4.03)	3.54 (2.94–4.25)	3.01 (2.50–3.61)	3.25 (2.68–3.93)	4.12 (3.26–5.20)	4.25 (3.33–5.41)
Emergency	2.20 (1.90–2.54)	3.15 (2.61–3.80)	2.62 (2.19–3.13)	2.99 (2.37–3.77)	2.41 (2.00–2.89)	3.32 (2.66–4.14)
Others	1.11 (0.95–1.29)	1.24 (1.06–1.44)	1.00 (0.85–1.18)	1.11 (0.94–1.32)	1.55 (1.26–1.89)	1.56 (1.28–1.92)
Average monthly volume						
Low	Ref	ref	ref	ref	ref	ref
Medium	1.05 (0.95–1.16)	1.00 (0.89–1.12)	0.80 (0.71–0.89)	0.88 (0.78–1.01)	1.16 (1.01–1.32)	1.04 (0.89–1.22)
High	1.10 (0.98–1.24)	0.86 (0.75–1.00)	0.81 (0.71–0.92)	0.80 (0.68–0.94)	1.19 (1.01–1.40)	0.84 (0.69–1.02)
Ownership of practice setting						
Private	Ref	ref	ref	ref	ref	ref
Public	0.63 (0.56–0.71)	0.80 (0.71–0.91)	0.87 (0.75–1.00)	0.91 (0.79–1.05)	0.62 (0.54–0.71)	0.82 (0.70–0.95)
Accreditation level of practice setting						
Medical center	ref	ref	ref	ref	ref	ref
Regional/District hospital	0.99 (0.87–1.13)	1.03 (0.92–1.16)	0.98 (0.84–1.14)	1.07 (0.92–1.23)	0.90 (0.77–1.05)	0.95 (0.82–1.10)
Clinics	1.32 (1.17–1.49)	1.38 (1.20–1.59)	0.98 (0.86–1.13)	1.21 (1.02–1.44)	1.33 (1.14–1.54)	1.28 (1.07–1.54)
Urbanization of practice						
Urban	ref	ref	ref	ref	ref	ref
Non-urban	1.16 (1.03–1.30)	1.10 (0.97–1.25)	1.07 (0.94–1.22)	1.06 (0.93–1.21)	1.10 (0.96–1.27)	1.08 (0.92–1.25)

$30 NTD ≌ $1 US dollar.

Abbreviation: SES, socioeconomic status.

After adjusting for patient characteristics, antibiotic prescribing in the outpatient setting varied significantly among physicians. Antibiotic prescribing increased with physician age. Compared to family medical physicians, Otolaryngology (OR: 3.54; 95% CI: 2.94–4.25), emergency (OR: 3.15; 95% CI: 2.61–3.80) and other specialists (OR: 1.24; 95% CI: 1.06–1.44) were significantly more likely to order antibiotics. Among practice settings, physicians in public hospitals were 20% less likely to prescribe antibiotics (95% CI: 0.71–0.91) than in private settings; at a facility level, antibiotics were most likely to be prescribed in clinics (OR: 1.38; 95% CI: 1.20–1.59) compared to all levels of hospitals.

The most commonly prescribed antibiotics for dengue outpatients were mainly cephalosporins (i.e. cefalexin, cefradine, cefazolin, cefadroxil, cefuroxime; 49.9%-55.4%) and tetracyclines (doxycycline; 4.8%-6.3%; Table 4).

**Table 4:** The top 5 antibiotics used in adult dengue patients.

	Outpatient Setting				Inpatient Setting				
	Pre-dengue confirmation		Post-dengue confirmation			Pre-dengue confirmation		Post-dengue confirmation	
No.	Antibiotics	%	Antibiotics	%		Antibiotics	%	Antibiotics	%
1	cefalexin	29.9	cefalexin	27.9		cefazolin	38.1	cefazolin	34.3
2	cefradine	14.2	cefradine	14.5		ceftriaxone	16.2	cefuroxime	13.8
3	clindamycin	7.9	cefazolin	7.3		levofloxacin	15.2	ceftriaxone	13.7
4	cefadroxil	5.8	doxycycline	6.3		doxycycline	15.0	levofloxacin	11.9
5	doxycycline	4.8	cefuroxime	5.7		cefuroxime	10.9	doxycycline	8.9

### Antibiotic prescribing in inpatient setting

Overall, 30.1% of inpatients without a concomitant bacterial infection were prescribed an antibiotic (Table 5). Proportions of inpatient admissions with antibiotic prescriptions varied remarkably across patient characteristics. Inpatients 65 years or older (40.4%), female inpatients (32.6%), and inpatients with major comorbidity (42.4%) received antibiotic prescriptions more frequently than their counterparts. Hospitalized patients in the epidemic years received antibiotic prescriptions less frequently (29.2%) than those in the nonepidemic period (35.4%).

**Table 5:** Inpatient antibiotic use for confirmed dengue by patient and physician characteristics, in pre- and post- dengue diagnosis periods, Taiwan, 2008–2015.

	Total		Pre-dengue confirmation			Post- dengue confirmation		
		Antibiotics use		Antibiotics use			Antibiotics use	
	N (%)	%	N (%)	%	P	N (%)	%	P
**Total admissions**	**18,629**	**30.1%**	**2,900**	**52.3%**		**15,729**	**26.0%**	
**Epidemic period**					.50			.30
No	2,703 (14.5)	35.4%	885 (53.1)	53.1%		1,854 (11.6)	26.8%	
Yes	15,926 (85.5)	29.2%	2,015 (52.0)	52.0%		14,194 (88.4)	25.9%	
**Patient characteristics**								
Age, y					< .01			< .01
18–45	5,418 (29.1)	23.2%	826 (46.2)	46.2%		4,661 (29.0)	19.0%	
45–64	6,581 (35.3)	25.3%	998 (46.2)	46.2%		5,684 (35.4)	21.6%	
≥65	6,630 (35.6)	40.4%	1,076 (62.6)	62.6%		5,703 (35.5)	36.1%	
Sex					< .01			< .01
Male	8,845 (47.5)	27.3%	1,379 (48.0)	48.0%		7,630 (47.5)	23.4%	
Female	9,784 (52.5)	32.6%	1,521 (56.2)	56.2%		8,418 (52.5)	28.3%	
SES					.04			< .01
< $30,000 NTD	6,395 (34.3)	28.3%	993 (52.1)	52.1%		5,499 (34.3)	24.0%	
≥ $30,000 NTD	6,218 (33.4)	28.4%	885 (48.8)	48.8%		5,427 (33.8)	25.1%	
Others	6,016 (32.3)	33.6%	1,022 (55.6)	55.6%		5,122 (31.9)	29.1%	
Major comorbidity					< .01			< .01
No	16,903 (90.7)	28.8%	2,567 (50.5)	50.5%		14,594 (90.9)	24.9%	
Yes	1,726 (9.3)	42.4%	333 (66.1)	66.1%		1,454 (9.1)	36.7%	
**Physician characteristics**								
Physician age, Y					.60			< .01
≤40	7,243 (38.9)	32.6%	1,144 (51.8)	51.8%		6,099 (29.0)	29.0%	
40–59	10,813 (58.0)	28.8%	1,675 (52.8)	52.8%		9,138 (24.4)	24.4%	
≥60	573 (3.1)	21.8%	81 (48.1)	48.1%		492 (17.5)	17.5%	
Physician sex					.67			.68
Male	16,251 (87.2)	29.9%	2,486 (52.4)	52.4%		13,765 (25.8)	25.8%	
Female	2,378 (12.8)	31.2%	414 (51.7)	51.7%		1,964 (26.8)	26.8%	
Average monthly volume					< .01			< .01
Low	6,369 (34.2)	33.0%	950 (57.7)	57.7%		5,419 (28.7)	28.7%	
Medium	5,995 (32.2)	25.9%	1,078 (47.6)	47.6%		4,917 (21.1)	21.1%	
High	6,265 (33.6)	31.1%	872 (52.3)	52.3%		5,393 (27.7)	27.7%	
Ownership of practice setting					.06			< .01
Private	10,849 (58.2)	28.8%	1,944 (50.8)	50.8%		8,905 (24.0)	24.0%	
Public	7,780 (41.8)	31.9%	956 (55.4)	55.4%		6,824 (28.6)	28.6%	
Accreditation level of practice setting					.74			< .01
Medical center	5,803 (31.2)	32.4%	877 (54.0)	54.0%		4,926 (28.6)	28.6%	
Regional/District hospital	12,826 (68.8)	29.0%	2,023 (51.6)	51.6%		10,803 (24.8)	24.8%	
Clinics								
Urbanization of practice					.05			< .01
Urban	16,032 (86.1)	30.3%	2,238 (53.4)	53.4%		13,794 (26.5)	26.5%	
Non-urban	2,597 (13.9)	29.0%	662 (48.8)	48.8%		1,935 (22.2)	22.2%	

$30 NTD ≌ $1 US dollar.

Abbreviation: SES, socioeconomic status; P, P-value.

In contrast, physician characteristics did not vary except for physician age: 32.6% ordered by younger physicians (< 40 years) compared to 21.8% by older physicians (60 years or above). Patients who were hospitalized after dengue was confirmed (26.0%) received antibiotic prescriptions less frequently than those hospitalized before confirmation (52.3%). Specific antibiotics ordered for patients hospitalized before and after the confirmation of dengue were similar.

After adjusting for other characteristics and clustering effects, prescribing patterns were relatively consistent for all inpatient admissions (Table 6). Likelihood of receiving an antibiotic increased with advancing age: >65 years old (OR: 1.88; 95% CI: 1.71–2.07), 45–64 years old (OR: 1.12; 95% CI: 1.05–1.20), compared to younger inpatients. Females (OR: 1.24; 95% CI: 1.18–1.31), and patients with a major comorbidity (OR: 1.38; 95% CI: 1.25–1.53) were significantly more likely to receive an antibiotic. Antibiotics were ordered less frequently in epidemic years (OR: 0.72; 95% CI: 0.62–0.84) than in the nonepidemic period. In terms of physician characteristics, physicians with a larger inpatient volume (OR: 0.73; 95% CI: 0.60–0.90) and those practicing in regional/district hospitals (OR: 0.56; 95% CI: 0.48–0.66) were significantly less likely to prescribe antibiotics to dengue inpatients than their counterparts. Cephalosporins (i.e. cefazolin, ceftriaxone, cefuroxime; 61.8%-65.2%), tetracyclines (i.e. doxycycline; 8.9%-15.0%), quinolones (i.e. levofloxacin; 11.9%-15.2%) were the most commonly prescribed antibiotics, accounting for more than 80% of all dengue inpatient courses. The classes of antibiotic prescribed before and after dengue diagnosis were similar.

**Table 6:** Patient and physician characteristics associated with antibiotic prescribing for dengue patients in inpatient setting, Taiwan, 2008–2015.

	Total		Pre-dengue confirmation		Post- dengue confirmation	
	OR (95%CI)	AOR (95% CI)	OR (95%CI)	AOR (95% CI)	OR (95%CI)	AOR (95% CI)
**Epidemic period**						
No	ref	ref	ref	ref	ref	ref
Yes	0.80 (0.69–0.91)	0.72 (0.62–0.84)	1.00 (0.82–1.21)	0.90 (0.75–1.09)	0.96 (0.82–1.12)	0.88 (0.74–1.04)
**Patient characteristics**						
Age, y						
18–45	ref	ref	ref	ref	ref	ref
45–64	1.15 (1.08–1.22)	1.12 (1.05–1.20)	1.01 (0.85–1.19)	0.98 (0.82–1.17)	1.17 (1.09–1.27)	1.14 (1.05–1.24)
≥65	1.93 (1.76–2.12)	1.88 (1.71–2.07)	1.77 (1.44–2.19)	1.76 (1.43–2.18)	2.00 (1.80–2.23)	1.90 (1.71–2.12)
Sex						
Male	ref	ref	ref	ref	ref	ref
Female	1.25 (1.19–1.32)	1.24 (1.18–1.31)	1.38 (1.20–1.58)	1.38 (1.19–1.60)	1.26 (1.18–1.33)	1.25 (1.18–1.33)
SES						
< $30,000 NTD	ref	ref	ref	ref	ref	ref
≥ $30,000 NTD	0.98 (0.92–1.04)	0.94 (0.88–1.00)	0.89 (0.75–1.05)	0.85 (0.72–1.00)	1.02 (0.95–1.09)	0.97 (0.90–1.04)
Others	1.18 (1.11–1.25)	1.02 (0.96–1.08)	1.06 (0.91–1.23)	0.91 (0.78–1.06)	1.21 (1.13–1.29)	1.04 (0.97–1.12)
Major comorbidity						
No	ref	ref	ref	ref	ref	ref
Yes	1.53 (1.40–1.69)	1.38 (1.25–1.53)	1.57 (1.24–1.98)	1.48 (1.17–1.88)	1.51 (1.36–1.68)	1.33 (1.19–1.48)
**Physician characteristics**						
Physician age, Y						
≤40	ref	ref	ref	ref	ref	ref
40–59	0.98 (0.87–1.10)	1.01 (0.89–1.14)	1.16 (0.93–1.45)	1.23 (0.98–1.55)	0.99 (0.86–1.13)	0.96 (0.83–1.11)
≥60	0.71 (0.50–1.03)	0.74 (0.52–1.06)	0.97 (0.53–1.77)	1.00 (0.53–1.88)	0.67 (0.42–1.06)	0.67 (0.43–1.05)
Physician sex						
Male	ref	ref	ref	ref	ref	ref
Female	0.92 (0.76–1.11)	0.90 (0.73–1.10)	0.97 (0.73–1.28)	0.99 (0.74–1.32)	0.95 (0.77–1.16)	0.91 (0.73–1.14)
Average monthly volume						
Low	ref	ref	ref	ref	ref	ref
Medium	0.88 (0.78–1.00)	0.90 (0.77–1.06)	0.73 (0.60–0.89)	0.73 (0.60–0.90)	0.85 (0.73–0.98)	0.88 (0.75–1.04)
High	0.95 (0.81–1.11)	1.02 (0.86–1.20)	0.93 (0.72–1.18)	0.93 (0.73–1.19)	0.91 (0.76–1.08)	0.97 (0.80–1.16)
Ownership of practice setting						
Private	ref	ref	ref	ref	ref	ref
Public	0.94 (0.81–1.08)	0.95 (0.83–1.09)	0.99 (0.80–1.24)	0.99 (0.78–1.26)	1.00 (0.84–1.18)	0.98 (0.85–1.14)
Accreditation level of practice setting						
Medical center	ref	ref	ref	ref	ref	ref
Regional/District hospital	0.64 (0.56–0.73)	0.65 (0.56–0.75)	0.97 (0.78–1.21)	1.03 (0.80–1.31)	0.54 (0.47–0.63)	0.56 (0.48–0.66)
Urbanization of practice						
Urban	ref	ref	ref	ref	ref	ref
Non-urban	0.96 (0.75–1.23)	1.23 (0.98–1.55)	1.02 (0.77–1.37)	1.12 (0.81–1.55)	0.80 (0.65–0.98)	1.12 (0.90–1.40)

$30 NTD ≌ $1 US dollar.

Abbreviation: SES, socioeconomic status; P, P-value.

## Discussion

This population-based study of antibiotic prescribing patterns for dengue in Taiwan between 2008–2015 found several associations of interest. Significantly, more than 1 in 4 dengue patients received at least one antibiotic prescription during the 14-day assessment period (i.e. from 7 days before to 7 days after dengue was confirmed). The proportion was significantly lower for both inpatients and outpatients in the two epidemic years than in nonepidemic years, indicating the underestimation of dengue in physicians’ pre-test assessments. Antibiotics were prescribed more frequently in inpatient (30.1%) than in ambulatory care settings (6.1%) with usage higher for older patients, females and patients with underlying conditions. In examining physician characteristics associated with antibiotic prescription, older physician age and private practice were significant factors influencing the decision to prescribe antibiotics in outpatient visits. The likelihood of prescribing an antibiotic to dengue patients was reduced by more than 50% in medical visits occurring after the dengue infection was confirmed.

Antibiotic prescribing for dengue in outpatient visits (6.1%) was not as widespread as that observed for common viral respiratory illnesses such as acute bronchitis in Taiwan [13,30,31], a fraction that also should be viewed in the context that dengue was listed as a diagnosis in only 8.7% of visits before the diagnosis was confirmed. The frequent use of antibiotics among hospitalized dengue patients is a potential concern: while antibiotics were ordered in 52.3% of patients before dengue was confirmed, that fraction dropped to only 26.0% after the diagnosis was confirmed. In this database analysis, we are unable to ascertain why antibiotics were continued or ordered after dengue was diagnosed but the observation suggests the need for further investigation. Our estimates may not be compared directly to the findings in other studies that described antibiotic prescribing or usage in acute febrile illness (AFI) patients, rather than specifically in confirmed dengue patients.

Consistent with previous findings [9,32], our study also showed significantly lower antibiotic prescribing for dengue during outbreak compared to baseline years. Physicians likely internalized the higher likelihood of dengue as the cause of febrile illnesses during this period, as the course of the outbreak and amplified dengue control and preventive efforts were well publicized. The distinction between epidemic and other seasons is sharper in Taiwan than in countries where dengue is endemic so this impact on clinical diagnosis and practice may not be generalizable.

Diagnostic uncertainty is a principal driver of physician decision-making in prescribing antibiotics [33]. For dengue, previous studies showed that a positive dengue test result can significantly reduce antibiotic prescribing for AFI patients, a trend was reflected in our analysis of both outpatients and inpatients [34]. The recommended approach for laboratory confirmation of infection necessitates testing an acute sample for viral RNA followed by testing serum days later to demonstrate the development of specific IgM, because the window for viremia may have been missed at the first visit [35]. Until improved rapid diagnostic assays are developed and made accessible to developing and low-income countries where dengue is transmitted in an endemic pattern, antibiotics will continue to be used unnecessarily to varying degrees [22,23,36,37]. The finding suggests that facilitating development and distribution of more timely and sensitive dengue diagnostic tools could reduce unnecessary antibiotic use for suspect dengue cases.

Consistent with previous studies, patient’s age, gender and comorbidity status play significant roles in prescribing decisions in both outpatient and inpatient care settings. As seen previously [38,39], females were significantly more likely to be prescribed an antibiotic. An increased likelihood of antibiotic prescribing for older hospitalized patients and those with other major comorbidities may reflect physician precautionary prescribing behaviors towards patients with greater disease severity or risk for severe complications [9,34].

Significant prescribing variations were observed among ambulatory care physicians across age and specialty, which may reflect differences in medical education and training of the respective cohorts. The trend for greater frequency of antibiotic prescribing with older age may be intuitive as attention to antimicrobial resistance in medical school education and residency training has been greater in recent decades. In addition to physician’s age and specialty, accreditation level and ownership of physician’s practice setting independently contributed to the decision. Physicians practicing in more technologically advanced hospitals were significantly more cautious in prescribing antibiotics than their counterparts in local clinics, possibly due to easier access to laboratory facilities, stronger demand for compliance to up-to-date guidelines, stricter continuing education requirements and peer pressure [13,30,40]. Poorer access to laboratory facilities may have driven more precautionary prescribing by clinic physicians. Another plausible explanation may be weaker links between physician earnings and sales of drugs in public or higher-level hospitals. Varying antibiotic prescribing practices in outpatient settings, but not in inpatient care, suggests the need to tailor antibiotic stewardship strategies [41–43].

Among the study limitations, as NHI claims data capture antibiotics prescribed under the NHI program, out-of-pocket purchases by patients could not be included, leading to an underestimation of antibiotic use. Secondly, although we excluded visits with a concomitant bacterial infection, as outpatient claims only allow up to 3 diagnoses, bacterial infection or other conditions for which antibiotics may be indicated may not have been captured fully, leading to an overestimation. Third, as we did not review individual medical records and due to data limitation of the NHI claims data, we lacked more detailed clinical information to ascertain appropriateness of and indications for antibiotic prescribing. Therefore, to be conservative, we excluded all visits with bacterial infection from our main analysis and solely focused on describing potentially unnecessary antibiotic prescribing among dengue cases. Future research with more detailed clinical data may help in this regard. Fourth, confounding bias may have been introduced as data on laboratory results, clinical presentation and symptoms, disease severity, and physician-patient communication were lacking, impeding detailed investigations into rationales behind physicians’ prescribing decisions. Finally, because the study was conducted in a setting under a national health insurance program with easy access to providers and prescription drugs, the results may not apply to countries with different levels of resources or different health care systems.

In conclusion, this population-based study of antibiotic prescribing for dengue patients systematically investigated a wide array of patient, physician, contextual factors and identified potentially modifiable circumstances that could be targeted for improved antibiotic stewardship. In Taiwan, at least, a “one-size fits all” antibiotic stewardship approach may not be effective in reducing unnecessary antibiotic prescribing for dengue. The aggregate result also provides a baseline for antibiotic use that hypothetically could be avoided when improvements in vector control and, potentially, introduction of effective vaccines reduce or even eliminate dengue transmission.

## Supporting information

S1 Text Supplementary tables. Table A in S1 Text. The average length of hospital stay among patients who had been hospitalized (N = 19,298). Table B in S1 Text. Patient and physician characteristics associated with antibiotic prescribing for dengue patients in outpatient settings, Taiwan, 2008–2015. Table C in S1 Text. Patient and physician characteristics associated with antibiotic prescribing for dengue patients in inpatient setting, Taiwan, 2008–2015.
(DOCX)
