# Supplementary material for: Population-based assessment of factors influencing antibiotic prescribing for adults with dengue infection in Taiwan
Source: PLoS Negl Trop Dis. 2022 Feb 28;16(2):e0010198. doi: 10.1371/journal.pntd.0010198 (PMC8884547; doi:10.1371/journal.pntd.0010198)
Supplement: S1 Text — Table A in S1 Text. The average length of hospital stay among patients who had been hospitalized (N = 19,298). Table B in S1 Text. Patient and physician characteristics associated with antibiotic prescribing for dengue patients in outpatient settings, Taiwan, 2008–2015. Table C in S1 Text. Patient and physician characteristics associated with antibiotic prescribing for dengue patients in inpatient setting, Taiwan, 2008–2015. (DOCX) [file pntd.0010198.s001.docx]

**Table A. The average length of hospital stay among patients who had been hospitalized (N=19,298).**

|  | **Antibiotics prescription** | |
| --- | --- | --- |
|  | **Yes** | **No** |
| **N** | 8,142 | 11,156 |
| **Length of hospital stay** | 8.1 (9.7) | 6.3 (5.5) |

**Table B. Patient and physician characteristics associated with antibiotic prescribing for dengue patients in outpatient settings, Taiwan, 2008-2015.**

|  | **Total** | |  | **Pre-dengue confirmation** | |  | **Post-dengue confirmation** | |
| --- | --- | --- | --- | --- | --- | --- | --- | --- |
|  | **OR (95%CI)** | **AOR (95% CI)** |  | **OR (95%CI)** | **AOR (95% CI)** |  | **OR (95%CI)** | **AOR (95% CI)** |
| **Bacterial Infections** |  |  |  |  |  |  |  |  |
| No | ref | ref |  | ref | ref |  | ref | ref |
| Yes | 1.04 (0.87 - 1.24) | 1.10 (0.92 - 1.33) |  | 0.81 (0.64 - 1.04) | 0.90 (0.70 - 1.16) |  | 1.24 (0.96 - 1.60) | 1.26 (0.97 - 1.65) |
| **Epidemic period** |  |  |  |  |  |  |  |  |
| No | ref | ref |  | ref | ref |  | ref | ref |
| Yes | 0.70 (0.65 - 0.76) | 0.67 (0.62 - 0.72) |  | 0.84 (0.77 - 0.92) | 0.81 (0.74 - 0.89) |  | 0.65 (0.58 - 0.73) | 0.60 (0.53 - 0.67) |
| **Patient characteristics** |  |  |  |  |  |  |  |  |
| Age, y |  |  |  |  |  |  |  |  |
| 18-45 | ref | ref |  | ref | ref |  | ref | ref |
| 45-64 | 0.89 (0.86 - 0.93) | 0.89 (0.85 - 0.93) |  | 0.87 (0.82 - 0.92) | 0.88 (0.84 - 0.93) |  | 0.90 (0.85 - 0.95) | 0.88 (0.83 - 0.94) |
| ≥65 | 0.97 (0.91 - 1.04) | 1.00 (0.93 - 1.06) |  | 0.86 (0.80 - 0.94) | 0.90 (0.84 - 0.98) |  | 1.03 (0.94 - 1.13) | 1.06 (0.96 - 1.17) |
| Sex |  |  |  |  |  |  |  |  |
| Male | ref | ref |  | ref | ref |  | ref | ref |
| Female | 1.12 (1.08 - 1.17) | 1.14 (1.09 - 1.18) |  | 1.06 (1.01 - 1.12) | 1.08 (1.02 - 1.13) |  | 1.20 (1.13 - 1.27) | 1.22 (1.15 - 1.30) |
| SES |  |  |  |  |  |  |  |  |
| < $30,000 NTD | ref | ref |  | ref | ref |  | ref | ref |
| ≥ $30,000 NTD | 0.95 (0.91 - 0.99) | 0.98 (0.94 - 1.02) |  | 0.94 (0.89 - 1.00) | 0.97 (0.92 - 1.02) |  | 0.96 (0.91 - 1.03) | 0.99 (0.93 - 1.06) |
| Others | 0.99 (0.95 - 1.03) | 1.01 (0.97 - 1.05) |  | 0.97 (0.91 - 1.03) | 1.01 (0.95 - 1.07) |  | 1.00 (0.94 - 1.07) | 1.02 (0.95 - 1.09) |
| Major comorbidity |  |  |  |  |  |  |  |  |
| No | ref | ref |  | ref | ref |  | ref | ref |
| Yes | 1.12 (1.04 - 1.20) | 1.17 (1.08 - 1.25) |  | 0.98 (0.89 - 1.08) | 1.04 (0.95 - 1.14) |  | 1.24 (1.11 - 1.38) | 1.28 (1.15 - 1.43) |
| **Physician characteristics** |  |  |  |  |  |  |  |  |
| Physician age, Y |  |  |  |  |  |  |  |  |
| ≤40 | ref | ref |  | ref | ref |  | ref | ref |
| 40-59 | 0.96 (0.86 - 1.07) | 1.06 (0.95 - 1.18) |  | 0.89 (0.79 - 1.01) | 1.07 (0.95 - 1.21) |  | 1.04 (0.91 - 1.19) | 1.08 (0.94 - 1.25) |
| ≥60 | 1.24 (1.08 - 1.41) | 1.59 (1.37 - 1.83) |  | 1.03 (0.89 - 1.20) | 1.46 (1.24 - 1.72) |  | 1.53 (1.28 - 1.84) | 1.84 (1.50 - 2.25) |
| Physician sex |  |  |  |  |  |  |  |  |
| Male | ref | ref |  | ref | ref |  | ref | ref |
| Female | 0.71 (0.61 - 0.82) | 0.86 (0.74 - 1.00) |  | 0.77 (0.65 - 0.91) | 0.89 (0.75 - 1.06) |  | 0.70 (0.58 - 0.84) | 0.87 (0.73 - 1.05) |
| Physician specialty |  |  |  |  |  |  |  |  |
| Family medicine | ref | ref |  | ref | ref |  | ref | ref |
| Internal medicine | 0.83 (0.71 - 0.96) | 0.95 (0.81 - 1.11) |  | 0.94 (0.79 - 1.12) | 1.00 (0.84 - 1.20) |  | 0.92 (0.76 - 1.12) | 1.00 (0.82 - 1.22) |
| Otolaryngology | 3.40 (2.85 - 4.05) | 3.55 (2.96 - 4.27) |  | 3.03 (2.52 - 3.64) | 3.27 (2.70 - 3.95) |  | 4.14 (3.27 - 5.23) | 4.27 (3.35 - 5.44) |
| Emergency | 2.23 (1.92 - 2.58) | 3.22 (2.67 - 3.88) |  | 2.66 (2.23 - 3.18) | 3.07 (2.43 - 3.86) |  | 2.44 (2.03 - 2.93) | 3.38 (2.71 - 4.23) |
| Others | 1.11 (0.96 - 1.29) | 1.24 (1.06 - 1.45) |  | 1.00 (0.85 - 1.18) | 1.12 (0.94 - 1.33) |  | 1.56 (1.27 - 1.91) | 1.57 (1.28 - 1.93) |
| Average monthly volume |  |  |  |  |  |  |  |  |
| Low | ref | ref |  | ref | ref |  | ref | ref |
| Medium | 1.04 (0.94 - 1.15) | 0.99 (0.88 - 1.11) |  | 0.79 (0.71 - 0.88) | 0.88 (0.77 - 1.00) |  | 1.15 (1.01 - 1.31) | 1.04 (0.89 - 1.21) |
| High | 1.10 (0.98 - 1.24) | 0.86 (0.74 - 0.99) |  | 0.81 (0.71 - 0.92) | 0.80 (0.68 - 0.94) |  | 1.19 (1.01 - 1.39) | 0.84 (0.69 - 1.02) |
| Ownership of practice setting |  |  |  |  |  |  |  |  |
| Private | ref | ref |  | ref | ref |  | ref | ref |
| Public | 0.64 (0.56 - 0.72) | 0.81 (0.71 - 0.91) |  | 0.87 (0.75 - 1.00) | 0.92 (0.80 - 1.06) |  | 0.62 (0.54 - 0.71) | 0.82 (0.70 - 0.96) |
| Accreditation level of practice setting | |  |  |  |  |  |  |  |
| Medical center | ref | ref |  | ref | ref |  | ref | ref |
| Regional/District hospital | 1.00 (0.88 - 1.14) | 1.03 (0.92 - 1.16) |  | 0.99 (0.85 - 1.15) | 1.07 (0.93 - 1.23) |  | 0.90 (0.77 - 1.06) | 0.95 (0.82 - 1.10) |
| Clinics | 1.34 (1.18 - 1.51) | 1.40 (1.22 - 1.61) |  | 1.00 (0.87 - 1.15) | 1.24 (1.04 - 1.46) |  | 1.33 (1.14 - 1.55) | 1.30 (1.08 - 1.56) |
| Urbanization of practice |  |  |  |  |  |  |  |  |
| Urban | ref | ref |  | ref | ref |  | ref | ref |
| Non-urban | 1.16 (1.04 - 1.30) | 1.10 (0.98 - 1.25) |  | 1.07 (0.94 - 1.22) | 1.06 (0.93 - 1.21) |  | 1.11 (0.96 - 1.27) | 1.08 (0.92 - 1.26) |

$30 NTD ≌ $1 US dollar.

Abbreviation: SES, socioeconomic status.

**Table C. Patient and physician characteristics associated with antibiotic prescribing for dengue patients in inpatient setting, Taiwan, 2008-2015.**

|  | **Total** | |  | **Pre-dengue confirmation** | |  | **Post- dengue confirmation** | |
| --- | --- | --- | --- | --- | --- | --- | --- | --- |
|  | **OR (95%CI)** | **AOR (95% CI)** |  | **OR (95%CI)** | **AOR (95% CI)** |  | **OR (95%CI)** | **AOR (95% CI)** |
| **Bacterial Infections** |  |  |  |  |  |  |  |  |
| No | ref | ref |  | ref | ref |  | ref | ref |
| Yes | 2.17 (1.72 - 2.74) | 1.86 (1.45 - 2.39) |  | 1.39 (0.87 - 2.21) | 1.19 (0.71 - 2.00) |  | 2.50 (1.94 - 3.22) | 2.14 (1.63 - 2.81) |
| **Epidemic period** |  |  |  |  |  |  |  |  |
| No | ref | ref |  | ref | ref |  | ref | ref |
| Yes | 0.80 (0.69 - 0.92) | 0.72 (0.62 - 0.84) |  | 0.98 (0.81 - 1.18) | 0.89 (0.74 - 1.07) |  | 0.96 (0.82 - 1.13) | 0.87 (0.73 - 1.04) |
| **Patient characteristics** |  |  |  |  |  |  |  |  |
| Age, y |  |  |  |  |  |  |  |  |
| 18-45 | ref | ref |  | ref | ref |  | ref | ref |
| 45-64 | 1.16 (1.09 - 1.23) | 1.12 (1.05 - 1.20) |  | 1.02 (0.86 - 1.21) | 1.00 (0.84 - 1.19) |  | 1.18 (1.10 - 1.28) | 1.14 (1.05 - 1.23) |
| ≥65 | 1.95 (1.78 - 2.13) | 1.88 (1.71 - 2.06) |  | 1.77 (1.44 - 2.17) | 1.76 (1.43 - 2.16) |  | 2.02 (1.82 - 2.25) | 1.89 (1.69 - 2.10) |
| Sex |  |  |  |  |  |  |  |  |
| Male | ref | ref |  | ref | ref |  | ref | ref |
| Female | 1.24 (1.18 - 1.30) | 1.25 (1.18 - 1.31) |  | 1.36 (1.19 - 1.56) | 1.37 (1.18 - 1.57) |  | 1.24 (1.17 - 1.32) | 1.25 (1.18 - 1.33) |
| SES |  |  |  |  |  |  |  |  |
| < $30,000 NTD | ref | ref |  | ref | ref |  | ref | ref |
| ≥ $30,000 NTD | 0.98 (0.92 - 1.04) | 0.93 (0.88 - 0.99) |  | 0.89 (0.76 - 1.06) | 0.86 (0.73 - 1.01) |  | 1.01 (0.95 - 1.08) | 0.96 (0.89 - 1.03) |
| Others | 1.18 (1.11 - 1.25) | 1.01 (0.96 - 1.07) |  | 1.06 (0.92 - 1.24) | 0.92 (0.79 - 1.07) |  | 1.21 (1.13 - 1.29) | 1.04 (0.97 - 1.11) |
| Major comorbidity |  |  |  |  |  |  |  |  |
| No | ref | ref |  | ref | ref |  | ref | ref |
| Yes | 1.55 (1.43 - 1.69) | 1.35 (1.23 - 1.48) |  | 1.51 (1.21 - 1.88) | 1.41 (1.12 - 1.77) |  | 1.54 (1.40 - 1.70) | 1.30 (1.17 - 1.44) |
| **Physician characteristics** |  |  |  |  |  |  |  |  |
| Physician age, Y |  |  |  |  |  |  |  |  |
| ≤40 | ref | ref |  | ref | ref |  | ref | ref |
| 40-59 | 0.96 (0.86 - 1.08) | 1.00 (0.88 - 1.13) |  | 1.16 (0.93 - 1.43) | 1.24 (0.99 - 1.54) |  | 0.99 (0.86 - 1.13) | 0.95 (0.82 - 1.11) |
| ≥60 | 0.74 (0.52 - 1.06) | 0.75 (0.53 - 1.07) |  | 0.97 (0.54 - 1.72) | 0.98 (0.54 - 1.78) |  | 0.70 (0.44 - 1.10) | 0.67 (0.43 - 1.05) |
| Physician sex |  |  |  |  |  |  |  |  |
| Male | ref | ref |  | ref | ref |  | ref | ref |
| Female | 0.89 (0.74 - 1.08) | 0.88 (0.73 - 1.08) |  | 0.95 (0.72 - 1.24) | 0.96 (0.73 - 1.27) |  | 0.92 (0.75 - 1.13) | 0.90 (0.72 - 1.12) |
| Average monthly volume |  |  |  |  |  |  |  |  |
| Low | ref | ref |  | ref | ref |  | ref | ref |
| Medium | 0.85 (0.75 - 0.97) | 0.87 (0.74 - 1.02) |  | 0.75 (0.61 - 0.92) | 0.75 (0.61 - 0.93) |  | 0.83 (0.72 - 0.95) | 0.85 (0.72 - 1.00) |
| High | 0.95 (0.82 - 1.10) | 1.00 (0.85 - 1.17) |  | 0.94 (0.74 - 1.20) | 0.94 (0.74 - 1.20) |  | 0.92 (0.78 - 1.09) | 0.96 (0.80 - 1.16) |
| Ownership of practice setting |  |  |  |  |  |  |  |  |
| Private | ref | ref |  | ref | ref |  | ref | ref |
| Public | 0.91 (0.79 - 1.06) | 0.94 (0.82 - 1.07) |  | 0.98 (0.79 - 1.21) | 0.98 (0.77 - 1.23) |  | 0.98 (0.82 - 1.17) | 0.97 (0.84 - 1.13) |
| Accreditation level of practice setting | |  |  |  |  |  |  |  |
| Medical center | ref | ref |  | ref | ref |  | ref | ref |
| Regional/District hospital | 0.63 (0.55 - 0.72) | 0.65 (0.56 - 0.75) |  | 0.96 (0.78 - 1.19) | 1.02 (0.80 - 1.29) |  | 0.54 (0.46 - 0.62) | 0.56 (0.48 - 0.66) |
| Urbanization of practice |  |  |  |  |  |  |  |  |
| Urban | ref | ref |  | ref | ref |  | ref | ref |
| Non-urban | 0.97 (0.77 - 1.23) | 1.25 (1.00 - 1.56) |  | 1.04 (0.78 - 1.38) | 1.14 (0.83 - 1.55) |  | 0.81 (0.66 - 0.99) | 1.14 (0.91 - 1.41) |

$30 NTD ≌ $1 US dollar.

Abbreviation: SES, socioeconomic status.
